# Rehabilitation needs and adherence among TAVR patients and their caregivers during digital home-based cardiac rehabilitation: a qualitative longitudinal study

**DOI:** 10.1186/s12912-026-04631-x

**Published:** 2026-04-06

**Authors:** Ying Ying Jia, Ting Wu, Zhi Ting Guo, Jian Ping Song

**Affiliations:** 1 Nursing Department, The Second Affiliated Hospital of Zhejiang University School of Medicine, Hangzhou, 310000 China; 2 Nursing Department, Zhejiang University School of Medicine, Hangzhou, 310000 China

**Keywords:** Digital health, Cardiac rehabilitation, Qualitative research, Experience, Adherence

## Abstract

**Background:**

While considerable research has examined the effectiveness of digital cardiac rehabilitation, few qualitative studies have explored the dynamic, evolving experiences of post-transcatheter aortic valve replacement (TAVR) patients and their caregivers at different time points during digital home-based cardiac rehabilitation. Longitudinal studies that adopt a dyadic perspective to capture phase-specific changes are especially scarce. This gap limits the development of targeted support strategies.

**Methods:**

A qualitative longitudinal study was conducted using semi-structured interviews at three time points: pre-discharge, one month, and three months after the digital intervention began. A reflexive thematic approach was used for data analysis. Participants were recruited from a tertiary hospital between January and March 2024.

**Findings:**

Twenty-two patient-caregiver dyads were included, resulting in 132 individual interviews. Three stage-specific themes were identified along the digital home-based cardiac rehabilitation trajectory: hope and apprehension (pre-discharge), life’s adversities (1 month post-intervention), and adherence divergence in rehabilitation (3 months post-intervention). Pre-discharge, participants expressed hope alongside concerns about disease progression, confusion about digital rehabilitation, and insufficient caregiver capacity. At 1 month, patients struggled with symptoms and reduced social activities, while caregivers faced care-needs mismatch, adverse emotions, inadequate guidance, inaccessible medical services, and life challenges. At 3 months, patients showed either negative responses to disease management or active coping with postoperative physical symptoms.

**Conclusions:**

The findings underscore the need for support strategies tailored to specific recovery phases to address dynamic challenges and optimize digital cardiac care for patients after TAVR and their caregivers. Clinically, providing digital device training before discharge can reduce confusion and improve preparedness. Nurse-led remote support one month after the procedure can help manage symptoms, rectify care mismatches, and improve service accessibility. Adjusting personalized rehabilitation plans at three months may further enhance adherence to rehabilitation protocols. This study’s longitudinal dyadic design addresses a current research gap by capturing the evolving experiences of both patients and caregivers across distinct recovery stages, thereby offering new insights for clinical practice.

**Clinical trial number:**

Not applicable.

**Supplementary Information:**

The online version contains supplementary material available at 10.1186/s12912-026-04631-x.

## Introduction

Aortic stenosis (AS) is prevalent among older adults, for whom transcatheter aortic valve replacement (TAVR) is now the standard treatment [1, 2]. Despite its effectiveness, TAVR leaves patients at high risk for complications, readmission, and poor outcomes, particularly in the immediate post-procedural period [3–5]. Cardiac rehabilitation is a cost-effective model delivered across inpatient, outpatient, and home settings [6–8]. The initial phase is crucial for post-TAVR patients due to their heightened vulnerability [9]. However, hospital-based cardiac rehabilitation remains limited in scope, and outpatient programs continue to struggle with low rates of referral, uptake, and completion [10, 11]. The American Heart Association consequently advocates for the global implementation of digital, home-based cardiac rehabilitation programs [12]. Most patients are discharged directly home following TAVR to continue their recovery. This transition from hospital to home, however, is often inadequately supported. Patients and their caregivers frequently report feeling unprepared for the challenges that follow. They commonly struggle with persistent physical symptoms, psychological distress, limited resources, and significant caregiving burdens [13–16].

A promising solution is home-based and nurse-led cardiac rehabilitation [17]. Integrating such an intervention into primary care can enhance the health-related quality of life for both post-TAVR patients and their caregivers [16]. This model usually includes rehabilitation assessments, interventions (such as medication, exercise, nutrition, psychological support, and smoking cessation), and follow-up [6]. However, the implementation is hindered by several factors, notably the limited availability of primary medical health service facilities and personnel [18]. A growing body of research has explored home-based cardiac rehabilitation and family caregiving in post-TAVR populations [19, 20]. However, rehabilitation adherence—a critical determinant of success—remains poorly understood, especially within the Chinese healthcare context [21–23]. Home-based cardiac rehabilitation adherence describes the extent to which a patient’s out-of-hospital behaviors—including medication use, dietary and exercise management, smoking cessation, and psychological self-care—conform to the rehabilitation plan established by their clinical team [24].

However, no studies have examined the dynamic needs and longitudinal adherence patterns of Chinese patients and their caregivers following TAVR, particularly within digital home-based cardiac rehabilitation during the hospital-to-home transition. This gap hinders the development of targeted and sustainable interventions. Consequently, this study aims to identify the needs of post-TAVR patients and their caregivers for digital home-based cardiac rehabilitation. It also seeks to explore the factors influencing their rehabilitation adherence. Furthermore, the study will examine how these needs and adherence patterns change at different time points across the transition from hospital to home. By identifying context-specific needs and adherence barriers, this work will inform the design of tailored digital home-based cardiac rehabilitation for this population. Ultimately, the findings aim to support a more effective hospital-to-home transition and improve long-term rehabilitation adherence and outcomes.

## Method

### Study design

This study is a longitudinal qualitative design that tracks the experiences of patient-caregiver dyads across three time points, combining descriptive analysis at each stage with longitudinal comparison of dynamic changes [25, 26]. Each participant underwent three structured interviews: (1) before discharge, (2) one month after the digital intervention began, and (3) three months after the intervention started. The pre-discharge interview aimed to capture patients’ and caregivers’ initial cognition of digital home-based cardiac rehabilitation, laying a foundation for targeted discharge preparation; The 1-month post-intervention interview targets the critical adaptation period when patients transition from hospital to digital home-based cardiac rehabilitation, helping identify early barriers that may affect adherence. It also bridges the gap between pre-discharge needs assessment and 3-month long-term adherence outcomes, ensuring timely optimization of the intervention to support sustained rehabilitation [27]; and the 3-month after the digital intervention starts interview was consistent with clinical guidelines recommending a minimum 3-month cardiac rehabilitation cycle, ensuring the collection of long-term adherence experiences throughout a complete rehabilitation phase. The first interviews were held in the hospital, while the second and the third were conducted online. The Consolidated Criteria for Reporting Qualitative Research Checklist was adopted to ensure consistency throughout the study [14, 28]. The research was reviewed and approved by the Institutional Review Board at the Ethics Committee of the Second Affiliated Hospital of Zhejiang University School of Medicine (approval number. I2023957) to ensure adherence with ethical guidelines. Informed consent was duly obtained from all participants. They were informed of the voluntary nature of their participation and their right to withdraw at any time without repercussions. All data were securely stored on a password-encrypted external drive. After data analysis was completed, the Tencent meeting videos and all raw data were permanently deleted to ensure participant privacy.

### Sample and setting

Post-TAVR patients and their caregivers were recruited as dyads from the coronary care units, the Second Affiliated Hospital of ZheJiang University, from January 2024 to March 2024. In this study, participants assigned to the intervention group received a digital, home-based cardiac rehabilitation program [29]. This qualitative study was conducted concurrently within the randomized controlled trial (RCT) described above, recruiting post-TAVR patient-caregiver dyads from the coronary care units at the Second Affiliated Hospital of Zhejiang University. In the parent trial, participants in the intervention group completed a standardized 12-week digital home-based cardiac rehabilitation program. Throughout this period, all participants engaged in continuous home exercise management, performing aerobic exercise twice weekly for 30–40 min per session. During the initial four weeks, they also received a structured multi-component intervention addressing motivational enhancement, supportive environment construction, risk factor management, and home symptom monitoring with follow-up education. The digital platform issued weekly automated reminders, while study nurses conducted monthly telephone follow-ups to provide guidance, support, and feedback. The complete intervention protocol, timeline and related activities are detailed in Appendix A [29]. As a qualitative substudy nested within this RCT, our investigation aimed to complement the trial’s quantitative outcomes by exploring patients’ adherence experiences, unmet needs, and influencing factors during home-based cardiac rehabilitation under the same digital intervention. This approach yields in-depth insight into how and why the intervention succeeded or fell short, captures patients’ subjective perceptions and lived experiences, and generates contextual understanding not accessible through quantitative data alone. These findings will inform the refinement and optimization of the digital home-based rehabilitation program for post-TAVR patients in future clinical practice. 

Purposive sampling was used to select participants from the experimental group of the concurrent randomized controlled trial. Sampling criteria included age, education level, household income, and residence to ensure the representativeness of the sample within the experimental group, while maintaining the consistency of the standardized digital intervention framework of the randomized controlled trial. Data saturation was assessed using a two‑stage verification approach: intra‑stage saturation and overall saturation. After coding data from the dyads, no new themes related to adherence to digital home‑based cardiac rehabilitation emerged, indicating that data saturation was reached. For intra‑stage saturation, every 5 dyad interviews were coded independently by two researchers; saturation was confirmed if no new themes appeared in the following 3 or more dyads and existing themes were adequately detailed. For overall saturation, the research team performed a cross‑stage integrative analysis using NVivo after all three interview stages. Overall saturation was verified when no new cross‑stage themes emerged and all stage‑specific themes were fully covered without omission.

**Fig. 1:**
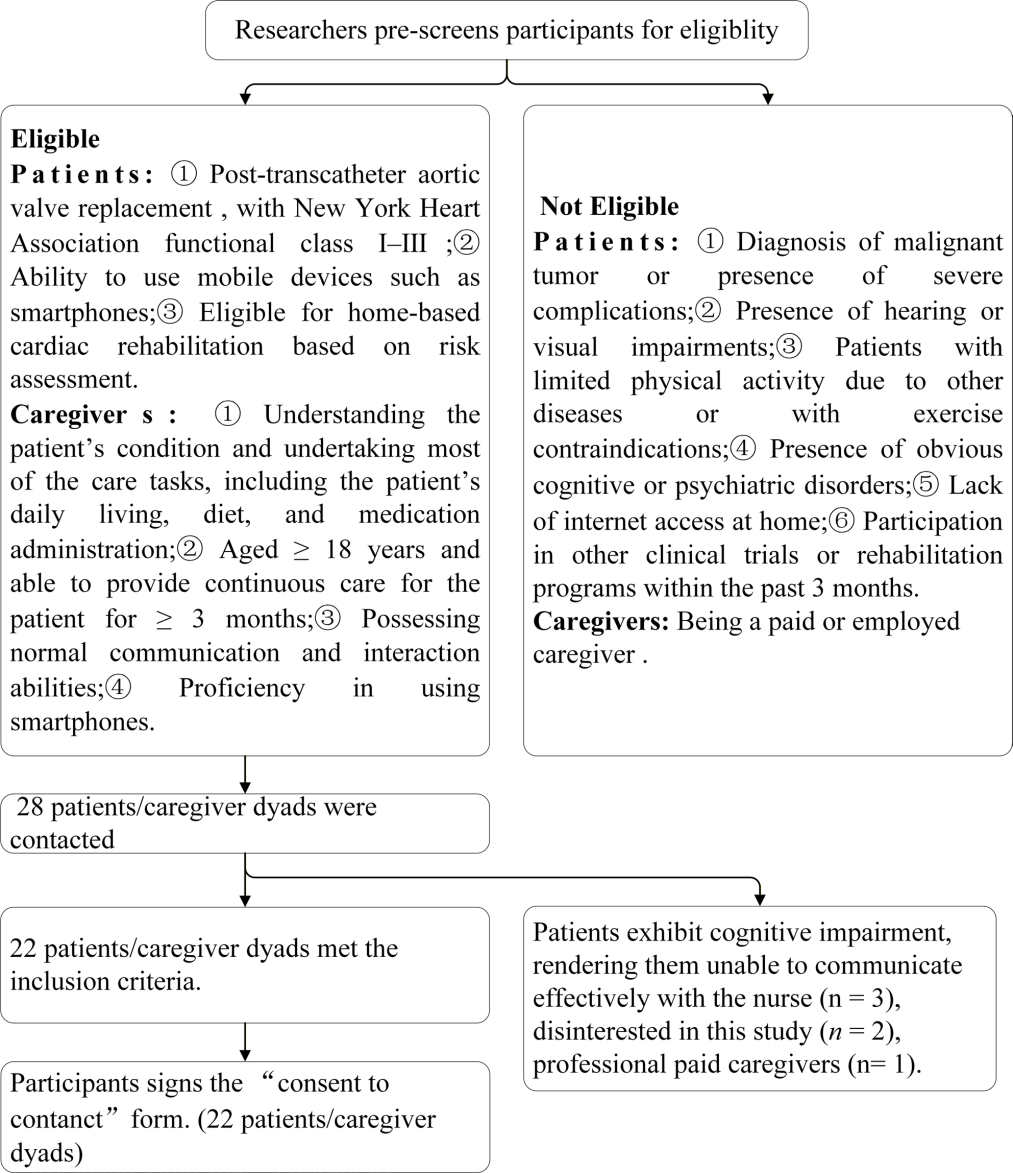
Recruitment pathway

All eligible patients met the inclusion criteria of our concurrent standardized digital home-based cardiac rehabilitation intervention study [29]. The recruitment process was conducted by two trained research nurses: during the patients’ hospitalization, the nurses first explained the purpose, interview schedule, data usage, and privacy protection measures of this qualitative study to the patients and their potential primary caregivers. Inclusion and exclusion criteria for patients are as detailed in Fig. 1.

### Procedure

An initial draft of the interview guide was developed based on a literature review. Prior to official interviews, two pre-interviews were conducted with three pairs of participants to refine the interview guide based on their feedback. The finalized interview guide is shown in Appendix B.

Information from participants interested in the project was recorded and forwarded to a project nurse for eligibility verification. The professional relationship existed between the researcher and all participants. The nurse explained to the eligible participants the details of the study, including the purpose, informed consent, and a brief demographic survey. These eligible participants were scheduled for individual interviews in a relaxed atmosphere. To minimize potential social desirability bias and the influence of professional relationships, an independent researcher not involved in the participants’ clinical care conducted all interviews. We guaranteed strict confidentiality and anonymity before each interview. Participants were explicitly informed that their responses would not affect their clinical treatment. During face-to-face interviews, the research team used a voice recorder to record the conversations. Online interviews were facilitated via private Tencent links. All participants were informed that the Tencent meeting videos would be recorded with field notes and deleted after data analysis.

A pilot interview with the first three dyads revealed that individual in semi-structured interviews effectively yielded comprehensive data and consensus themes. Moreover, family caregivers were far more willing to share the practical difficulties and hands-on experiences they encountered in daily caregiving when interviewed without the presence of patients. In this study, patients and caregivers were interviewed separately, with the core aim of ensuring that both parties could express their true views openly, especially those sensitive perspectives that are inconvenient to disclose in routine care settings. The recruitment process was conducted by two trained research nurses (YYJ and WT), a PhD candidate and a Master’s candidate in Nursing, respectively, both of whom had experience in qualitative research. The interviewers had no explicit bias or preset assumptions about TAVR patients’ and caregivers’ adherence in digital home-based cardiac rehabilitation; their research interest stemmed from the challenges of digital home-based cardiac rehabilitation in post-cardiac surgery populations and the lack of qualitative exploration of dyadic adherence.

The initial interviews were held in distinct wards and conference rooms within the hospital, involving the emotions, needs, and plans of post-TAVR patients and their caregivers prior to discharge. The second set of interviews were carried out online, covering participants’ needs for cardiac recovery, factors affecting caregiving, and obstacles. The final set of interviews was carried out online. This stage emphasized the factors affecting their adherence to home-based cardiac rehabilitation. Audio recordings were kept confidential and not linked to participant identities to ensure privacy. The duration of each interview was controlled between 30 and 60 min. No individuals other than the participants and the research team were present, so as to protect participants’ privacy, avoid external interference, and ensure they could freely express their experiences regarding adherence to digital home-based cardiac rehabilitation.

### Data analysis

NVivo software was applied for data management in this study. The corresponding author trained all researchers in qualitative analysis and supervised the process. Within 24 h of interview completion, two authors (YYJ and TW) transcribed the recordings into both Chinese and English. Subsequently, team members (JPS and ZTG) reviewed each transcript to verify accuracy.

Four team members attained familiarity with the content through repeated readings. According to the research objectives, each transcript was annotated with qualitative descriptive codes. Team members independently coded the highlighted data, which were then collated into analogous codes or relabeled as novel codes. The codes evolved dynamically across the three follow-up stages: initially focusing on pre-discharge cognition and needs, then shifting to practical challenges during the 1-month intervention, and finally concentrating on adherence-related responses at 3 months. For the longitudinal comparison, we used NVivo to construct a “code-stage-theme” matrix, tracking changes in code frequency and connotation across stages. This process ensured the rigor of the longitudinal analysis by clearly demonstrating the sequential development and logical connection of codes over time. Ultimately, a conclusive report was derived from debate, scrutiny, and articulation of distilled themes and their corollaries that mirrored the interview objectives. No themes were identified in advance; all core themes and sub-themes were derived from the transcribed interview data of post-TAVR patients and their caregivers, following a data-driven approach and reflexive thematic analysis.

### Quality control

Data trustworthiness was upheld throughout the iterative analysis process by underscoring credibility, confirmability, transferability, and dependability [30]. Interviews were conducted in secure and confidential environments to facilitate open communication, guaranteeing credibility. Patients and their caregivers were interviewed separately. All discussions were recorded and transcribed by professionals for accuracy. To ensure no sample loss across the three interview rounds, strict eligibility screening was implemented during recruitment. During follow-up, retention was enhanced through advance appointments, flexible adjustment of online interview times to fit caregivers’ schedules, and dedicated project nurses responsible for regular reminders and communication. A predefined protocol was adopted for potential partial data loss (e.g., recording interruptions): key information would be supplemented via on-site field notes, with participants contacted by phone within 24 h to confirm missing content, ensuring data integrity.

Additionally, credibility was reinforced by using four trained coders for data analysis under the supervision of a senior coder (JPS). The translated interpretations were cross-checked against the original recordings and transcripts. The reflexive thematic analysis was conducted on transcribed data as outlined by Braun and Clarke [31]. The data analysis employed a “data-driven approach” to elucidate and generalize the distinctive experiences of post-TAVR patients and their family caregivers during home-based cardiac rehabilitation [31]. After formulating the preliminary interview report, the report was sent to the interviewees to verify whether the content of the analysis was consistent with the original meaning the interviewees intended to express. To ensure coding reliability during thematic analysis, two independent researchers first coded 20% of the interview transcripts using the preliminary coding framework derived from the study objectives. Regarding researcher reflexivity, all researchers involved in data collection and analysis maintained reflexive manuscripts throughout the study, documenting their prior experiences with cardiac rehabilitation research, potential biases, and evolving perspectives during coding.

## Result

### Participant sociodemographic data

A total of 28 patient-caregiver dyads were approached (Fig. 1). Twenty-two consenting patient-caregiver dyads were recruited. Interview transcripts were independently coded and thematically cross-validated by two researchers. No new themes related to adherence to digital home-based cardiac rehabilitation emerged during the analysis, confirming data saturation and an adequate sample size. During the three interview rounds of this study, there was no sample loss. Twenty-two patient-caregiver dyads were included, with each dyad completing 3 individual interviews (pre-discharge, 1-month post-intervention, 3-month post-intervention), resulting in a total of 132 individual interviews. The interview duration ranged from 34 to 59 min, with a median of 45 min.

Table 1 outlines the characteristics of patient-caregiver dyads. For patients: (1) age ranged from 67 to 83 years and all had stable vital signs post-TAVR (2–4 weeks post-surgery). For caregivers: (1) age ranged from 42 to 70 years; (2) 18 (81.81%) were patients’ children, 4 (18.20%) were spouses.

**Table 1:** The demographics of patient-caregiver dyads

Characteristics	Number
Patient’s educational level	
Basic education (Primary school)	7
Secondary education (Junior high school and high school)	13
Higher education (Bachelor’s degree)	2
Age of patients	
<70	3
≥ 70	19
Age of patient’s caregivers	
< 50	9
≥ 50	13
Experience in patient care (caregivers)	
Yes	20
No	2

### Qualitative finding

Participants described their experience with home-based cardiac rehabilitation as evolving through distinct phases. Interconnected themes and subthemes emerged at each phase. The quotes were labeled “P” for “post-TAVR patients” or “C” for “caregivers,” followed by the participant number. Developed based on the study objective (exploring adherence to digital home-based cardiac rehabilitation) and preliminary thematic analysis, the tree included 3 main themes and 10 corresponding sub-themes, each linked to specific coding nodes derived from interview content. Participants transitioned from the discharge preparation stage (preparatory hopes and concerns) to the one-month intervention stage (practical life challenges) and finally to the three-month stage (divergent rehabilitation adherence), illustrating the longitudinal progression of their experiences. The needs, barriers, facilitators, and changes in adherence during the three phases of home-based cardiac rehabilitation in post-TAVR patients are summarized in Table 2.

**Table 2:** Home-based cardiac rehabilitation experiences among post-TAVR patients

Phase	Needs	Facilitators	Barriers	Impact on adherence
Discharge preparation stage	Clear rehabilitation guidance; Safe disease management; Digital device training; Caregiver education	Symptom relief after TAVR; Convenient digital rehabilitation; Individualized discharge guidance	Uncertainty about long-term cardiac function; Digital operation difficulties; Patient/caregiver confusion on rehabilitation	Unstable adherence readiness; Initial positive intention with high anxiety
One month after intervention	In-person guidance; Symptom management; Social support; Caregiver training; Accessible medical services; Economic/psychological support	Remote monitoring; Basic discharge education	Persistent symptoms; Reduced social activities; Care mismatch; Inaccessible follow-up; Caregiver burden; Family conflict; Economic pressure	Unstable adherence; High dropout risk; Reduced exercise frequency
Three months after intervention	Individualized/interesting rehabilitation; Continuous professional support; Long-term caregiver support	Proactive self-management; Healthy behaviors; Mobile communication with medical staff	Perceived rehabilitation ineffectiveness; Monotonous training; Passive disease attitude; Caregiver fatigue	Divergent adherence: active engagement vs. poor compliance/discontinuation

### Theme 1: Discharge preparation stage: Hopes and concerns regarding the upcoming launch of rehabilitation

The initial phase of home-based cardiac rehabilitation involved stabilized patients who had commenced inpatient rehabilitation. This stage was characterized by mixed emotions among post-TAVR patients and their caregivers prior to discharge, with three prominent subthemes identified.

#### Sub- theme 1: Hope and concern about disease development

Most patients and caregivers expressed strong optimism post-TAVR, as preoperative symptoms were substantially relieved. One participant noted, *“I’m so lucky. My surgery was successful. My previous chest tightness seems to have disappeared. The doctor said that I could live normally in the future. I’m very happy (P15*,* 81 years).”* However, patients frequently reported fear and uncertainty about long-term cardiac function and postoperative safety. For example, some participants questioned, *“Can I engage in strenuous household chores? Is it permissible for morning exercises? Is there a risk of sudden death? (P1*,* 78 years).”*

#### Sub- theme 2: Advantages and shortcomings of digital home-based rehabilitation

- Advantages

*Patients valued the convenience of digital home-based rehabilitation*,* as highlighted by one participant: “I think postoperative home-based cardiac rehabilitation is very convenient*,* allowing me to do rehabilitation training and monitoring at home and avoid frequent hospital visits (P4*,* 72 years).”*

(b) Shortcomings

*Older patients faced multiple challenges with digital home-based rehabilitation*,* primarily related to device operation and information accessibility.* As patient described, *“I worry that I will not be able to operate the digital devices correctly*,* which seems too challenging for me. I am afraid this might delay my recovery and eager for skill training on these devices (P12*,* 69 years).”*

#### Sub- theme 3: Patients’ confusion and caregivers’ insufficient knowledge and skills on home-based cardiac rehabilitation

- Patients’ confusion

Despite receiving tailored rehabilitation and feeling optimistic, some patients remained confused about understanding and implementing their rehabilitation plans. *“Do I need to start cardiac rehabilitation after being discharged? This is the first time I’ve heard of it.” “Although the nurse explained a lot to me*,* I still don’t know how to proceed (P1*,* 78 years).” “Although the manual is written in Mandarin*,* it is completely incomprehensible to me. I reviewed it but still do not know how to proceed. Videos or more accessible materials would help (P9*,* 74 years).”*

(b) Inadequate capacity of caregivers

Caregivers also reported confusion about post-TAVR recovery management and digital tool use. *“I am concerned about correctly using these digital rehabilitation tools at home. We lack experience and might find it confusing. Additionally*,* should the patient’s diet and medication be given special attention? We are uncertain about how to adjust the diet to align with the new rehabilitation plan (C21*,* 50 years).”*

### Theme 2: One month after the digital intervention starts: life’s challenges

In the second phase, participants began to navigate home-based rehabilitation and improve its quality. Five significant themes emerged in this phase.

#### Sub- theme 1: Struggles with symptoms

Most post-TAVR patients experienced ongoing symptomatic struggles, reduced independence, and concerns about heart valve function and mortality. *“I often feel very weak*,* and my memory is not very good. I dare not go out alone without someone accompanying me (P8*,* 79 years).”*

#### Sub- theme 2: Decrease in social activities

Long-term debilitation reduced patients’ social activities, leading to emptiness and diminished rehabilitation motivation. 12 of these 16 patients missed ≥ 2 days of weekly exercise, as they reported *“No one to encourage me to keep going (P5*,* 74 years).”* Limited mobility, lack of familiarity with smart devices, poor physical conditions, and a busy primary caregiver diminish opportunities for social engagement during home-based cardiac rehabilitation. *“I live alone in the countryside*,* my children all live in the city*,* and I usually spend time with my husband. I don’t have many friends*,* which makes me feel empty and monotonous (P5*,* 74 years).”*

#### Sub- theme 3: Mismatch between care and needs and adverse emotional reactions

Patients reported misalignment between their care needs and the care received, often feeling neglected by family caregivers. *“I have several sons*,* but they are not willing to take care of me now; it is a burden for them (P1*,* 78 years). They think my thoughts are outdated and cannot communicate well with caregivers (P8*,* 79 years).”*

#### Sub- theme 4: Lack of instructions and inaccessible medical services

Many patients reported uncertainty about performing fine motor skills and adjusting exercise intensity during rehabilitation, preferring in-person guidance. One patient stated: *“I have been hospitalized several times. Although nurses advise me to exercise after discharge*,* I am still unclear on how to do it (P3*,* 73 years).”* Patients also lacked clarity on self-monitoring procedures and how to seek professional help. *“My blood pressure readings significantly vary between morning and evening. I am not sure about the correct method for measuring it. However*,* I am unsure of how to seek help from a doctor or nurse (P7*,* 75 years).”*

While two-thirds of patients received immediate in-person guidance and attended one-month post-surgery follow-ups, others missed appointments due to poor healthcare infrastructure, uneven resource distribution, and travel barriers. *“I dare not go to the big city alone for medical treatment*,* and I don’t want to trouble my busy family (P2*,* 72 years).”*

#### Sub- theme 5: Challenges for caregivers

- Complicated disease management

Most family caregivers lacked professional training, especially when managing patients with multiple comorbidities. Beyond medication, diet, exercise, personal care, and safety monitoring, many struggled to balance caregiving with other life responsibilities. They expressed strong needs for high-quality remote follow-up and rehabilitation training. As one caregiver stated: *“Although the doctor provided a detailed home rehabilitation manual*,* I sometimes feel uncertain about how to follow the plan (C20*,* 62 years).”*

(b) Anxiety and fear experienced by caregivers

Time conflicts and limited health literacy contributed to poor understanding of discharge instructions and high caregiver stress, especially during symptom worsening. As a caregiver shared: *“After my father is discharged*,* I feel pressure and anxiety about his care because I am responsible for looking after him. I am very worried that I have not provided adequate care (C9*,* 50 years).” “Additionally*,* the long hours spent caring for the patient leave me physically and emotionally exhausted. Sometimes*,* I feel isolated and helpless (C3*,* 46 years).”*

(c) Deterioration of family relationships and disruption of interpersonal lives

Caregivers described role shifts, increased burden, and strained family bonds due to patients’ behavioral changes such as irritability, verbal abuse, and frequent complaints. *“I oversee all his activities*,* including eating*,* brushing his teeth*,* bathing*,* taking medication*,* and using the restroom. Since my mum was sick*,* she has become very irritable*,* especially after returning home. I engage in monotonous nursing work every day*,* which is quite distressing. Often*,* the entire morning is consumed with her care*,* leaving me no time for my own activities.” It feels as though he is no longer my mother but merely exists in a state of life* (C17, *42 years*).”

(d) Economic burdens

Financial strain was a major stressor for both patients and caregivers, affecting mood, recovery, rehabilitation engagement, and access to community services. *“I have made every possible effort to raise funds for my father’s surgery. However*,* I truly cannot afford to go to the hospital for a follow-up examination one month before and after the procedure. We lack additional funds for post-discharge rehabilitation (C5*,* 69 years).” “It’s normal for older patients at my age to get sick*,* and some illnesses are incurable. Medical expenses might just be a waste of money*,* and I don’t want to burden my children any further. At this age*,* I feel like I’ve lived enough (P5*,* 74 years).”*

### Theme 3: Three months after the intervention starts: adherence divergence in rehabilitation

#### Sub- theme 1: Negative responses to disease management

Patients showed divergent rehabilitation adherence. Some held passive attitudes, attributing their condition to age, genetics, or inevitable disease progression, and saw cardiac rehabilitation as ineffective. Low motivation and poor adherence were common. Many also found rehabilitation monotonous. *The current rehabilitation plan has become boring after following it for several months. Exercising alone at home is not enjoyable (P12*,* 69 years).”*

Some caregivers also struggled to support home‑based rehabilitation due to fatigue and competing responsibilities. *“I am already very busy working at the factory every day and simply don’t have the energy to manage home-based rehabilitation (C2*,* 50 years).”*

#### Sub- theme 2: Actively handling postoperative physical symptoms

- Seeking support from professionals

Many patients actively consulted medical staff for health education and tailored guidance to improve postoperative outcomes. *“I hope my rehabilitation plan can be adjusted promptly*,* and I will actively contact the doctor through mobile phone (P3*,* 73 years).”*

(b) Promoting healthy behaviors

Other patients adapted to chronic illness through proactive self-management, sustained adherence, and engagement in healthy daily activities. *“Every morning*,* I go to the park to practice Tai Chi*,* and I also do some light household chores during the day (P6*,* 79 years)”.*

## Discussion

The study provided a nuanced understanding of the experiences of patients with TAVR and their caregivers during digital home-based cardiac rehabilitation in China. Recovery from TAVR is a multifaceted process involving interrelated dimensions, including physical, psychological, and social support. This study examines how the dynamic interplay between patient and caregiver needs and available resources shapes the longitudinal evolution of rehabilitation experiences.

The study demonstrated that the discrepancy between expectations and reality in digital home-based rehabilitation is rooted in the unique physiological, cognitive, and contextual characteristics of patients with TAVR, a predominantly older population with limited digital literacy, age-related physical impairments, and inadequate prior exposure to digital health tools. This finding is consistent with previous qualitative evidence showing that limited illness knowledge, misinterpretation of symptoms, and the influence of family dynamics can significantly shape health behaviours in cardiovascular conditions. In women following acute myocardial infarction, delays in seeking care were strongly associated with difficulties in recognising the severity of the condition, competing family roles, and low perceived personal risk, highlighting how behavioural responses are embedded within a relational and socio-cultural context [32]. Similarly, in the present study, patients’ and caregivers’ uncertainty regarding rehabilitation tasks and disease management reflects a broader process in which health literacy, illness perception, and family support interact to influence engagement in care. These findings reinforce the need for tailored, dyadic, and phase-specific educational interventions led by nurses. In this study, uncertainty among patients and caregivers regarding rehabilitation tasks and disease management reflects a broader process in which health literacy, illness perception, and family support interact to shape engagement. These findings underscore the necessity for nurse-led educational interventions that are tailored, dyadic, and phase-specific. Our study extends the existing literature by identifying digital-specific barriers not fully addressed in Yuan L et al.’s broader predictor model, highlighting a critical gap in current digital health interventions for TAVR patients: the general failure to integrate geriatric-centered design, personalized support systems, and culturally accessible features into rehabilitation platforms [33]. Beyond emphasizing the need for pre-discharge education [34], this disparity underscores that healthcare providers must adopt a multidimensional intervention framework tailored to the study’s identified barriers—one that combines tailored technical training, psychological counseling, and ongoing post-discharge technical support [35]. From a nursing practice perspective, this framework offers actionable guidance for clinical nurses to provide targeted care. It supports optimizing home-based cardiac rehabilitation platforms through user-centric design modifications and integrated social support features to reduce social isolation. The framework also facilitates implementing phase-targeted interventions that address both general predictors and challenges specific to digital health delivery [36, 37]. This discrepancy peaks during early post-discharge follow-up and directly influences fluctuations in three-month adherence [38].

The study demonstrated that there was a gap in patients’ management of their symptoms and engagement in social activities within the current digital home-based cardiac rehabilitation offerings. Among factors influencing patients’ adherence to exercise, social interaction opportunities are significant [39]. This is consistent with previous research findings that peer support is an important measure to improve patients’ adherence to home-based exercise [35]. To strengthen the program’s social dimension—a key contribution of nursing practice—plans should retain synchronous elements and explore ways to increase participant interaction. This approach directly addresses the barrier of social isolation identified in our study and aligns with nursing’s priority of holistic patient care [40]. In addition, patients frequently encounter a deficiency of information and medical services during their recovery process, which hampers their ability to effectively manage symptoms and psychological stress. These challenges echo previous studies highlighting the importance of personalized care plans and the need for accessible, responsive support systems [41].

Another important finding of this study is that both patients and their caregivers are very concerned about the occurrence of cardiovascular events during home recovery [7, 8]. Due to insufficient primary healthcare services and staff, and caregivers being occupied with work, some patients feel they may face significant risks when doing exercise rehabilitation at home [42]. In addition, most patients are also very concerned about the lifespan of the valve, this is consistent with the research findings of Yaqoob, A et al. [43]. Current evidence indicates that the estimated lifespan of TAVR valves in many patients is approximately 5 to 6 years [44]. Research shows that smartwatches can promote remote healthcare for patients discharged after TAVR, enabling new remote follow-up strategies [45]. For nursing practice, this underscores the value of integrating wearable devices into digital rehabilitation programs to deliver real-time guidance and adaptive support, which addresses patient safety concerns and enhances nursing-led remote care.

Research also revealed that caregivers often endure the dual pressures of providing care and managing the emotional needs of patients, highlighting an often-overlooked aspect of cardiac care. The emotional and physical stressors faced by caregivers can profoundly impact their ability to provide effective support to patients. The central role of caregivers observed in this study confirms that recovery after major cardiovascular procedures is not an individual process but a shared and relational experience. Similar findings have been reported in critical care contexts, where family members described profound emotional involvement, role redefinition, and a need for structured support to make sense of the illness trajectory and their caregiving responsibilities [46]. Consequently, recognizing caregivers as active partners in rehabilitation, not passive supporters, is essential for improving adherence, emotional adjustment, and care continuity. This perspective further validates the dyadic longitudinal design employed in the present study. Future programs should integrate caregiver support initiatives, recognizing their crucial role in the rehabilitation process and ensuring they are also adequately supported.

By three months after the intervention starts, adherence to the rehabilitation program varies, influenced by the patients’ responses to ongoing symptoms and their coping strategies, this is consistent with previous research findings [23]. The study found that there are many barriers affecting patients’ adherence to rehabilitation during their home convalescence, such as insufficient nursing and medical resources, limited social support. This indicates that individual differences during the rehabilitation process and adaptability to the rehabilitation plan are pivotal factors. This finding underscores the need to dynamically adjust rehabilitation plans in nursing practice according to each patient’s specific circumstances. Such personalization can be achieved through more frequent nurse-led follow-ups and responsive feedback mechanisms. These tailored strategies enhance recovery effectiveness and improve patient satisfaction [41].

Methodologically, the longitudinal and dyadic design of this study constitutes a key strength and a unique contribution to the literature. In contrast to most prior TAVR rehabilitation research, which relies on cross-sectional data or single-perspective analyses, our longitudinal follow-up from pre-discharge to three months post-intervention captures dynamic shifts in needs and challenges over time. Simultaneously, the dyadic focus on both patients and their caregivers reveals the interdependent nature of their experiences. This approach enables the identification of phase-specific barriers that would remain obscured in cross-sectional or single-perspective studies. Consequently, it provides a more comprehensive understanding of the factors influencing adherence, thereby addressing a methodological gap and enhancing the validity of our findings.

Our study identified inaccessible medical services, travel difficulties, and limited primary healthcare resources as key barriers to adherence in home-based cardiac rehabilitation. These results also underline that adherence to home-based cardiac rehabilitation is strongly influenced by contextual and organisational factors. Qualitative research conducted among individuals with chronic conditions living in geographically disadvantaged areas has shown that limited access to healthcare services, social isolation, and the need to travel long distances for follow-up significantly affect engagement in care and perceived quality of life [46]. In line with these findings, the barriers reported by our participants suggest that digital rehabilitation programmes cannot be effective without the parallel strengthening of community-based services and accessible follow-up pathways.

Overall, the investigation into the experiences of patients and caregivers in digital home-based rehabilitation yields actionable, phase-specific insights: For the pre-discharge stage, deliver dyadic hands-on training for digital devices and simplified visual educational materials alongside pre-discharge question clarification sessions to address initial confusion and concerns; for the 1-month post-intervention stage, launch a nurse-led hotline for real-time symptom and technical support, link with community health centers for on-site follow-up, and integrate peer/caregiver support forums into the digital platform to mitigate isolation and care burdens; for the 3-month post-intervention stage, tailor rehabilitation plans and provide caregiver respite services and skill refresher training; at the policy level, fund community rehabilitation resource expansion, integrate wearable devices for real-time monitoring, and standardize TAVR-specific digital rehabilitation content and delivery to ensure consistency across settings.

The three main themes and their subthemes identified in this study are intrinsically linked to core components of adherence, including motivation, capability, digital literacy, access to services, and social support [47]. During the discharge preparation stage, confusion about rehabilitation plans and insufficient caregiver capacity (Subtheme 3) directly undermined patients’ ability to adhere to the regimen, while limitations of digital rehabilitation tools (Subtheme 2b) reflected inadequate digital literacy and limited opportunities for effective engagement [48]. Over the one-month intervention period, symptom distress (Subtheme 1) and lack of clear medical guidance (Subtheme 4) weakened patients’ adherence motivation. Meanwhile, reduced social activities (Subtheme 2) and caregiver challenges (Subtheme 5), including financial burden and role strain, diminished the social support and resources required for sustained adherence [49]. At the three-month stage, low adherence motivation was associated with negative responses to disease management (Subtheme 1). By contrast, patients who actively managed postoperative symptoms (Subtheme 2) showed improved capability and stronger motivation for rehabilitation adherence in this study. This positive shift was supported by professional guidance and health behavior promotion [50].

### Implications for practice

For clinical nursing, practitioners should address core patient and caregiver needs through dyadic digital device training, phase-targeted guidance, and dedicated caregiver support. At the organizational level, healthcare institutions ought to establish multi-level support systems, enrich the social support functions of digital platforms, and strengthen collaboration between tertiary hospitals and community health centers. Regarding health policy, relevant departments should allocate funding to expand community resources, formulate standardized TAVR rehabilitation guidelines, and support the integration of digital tools into medical insurance. A schematic synthesis of phase-specific interventions encompasses pre-discharge dyadic training and education, one-month nurse-led real-time support with community follow-up, and three-month personalized rehabilitation adjustment with caregiver refresher training, thereby enhancing practical applicability.

### Limitations

First, the single-center design, which recruited participants exclusively from a tertiary hospital in Hangzhou, may introduce selection bias. Most patients originated from economically developed regions with relatively high socioeconomic status and digital literacy, limiting the generalizability of the findings. Second, the longitudinal design’s repeated interviews could induce reactivity bias, as participants might modify their responses or behaviors upon knowing they were being observed. Third, conducting some interviews online may have diminished the richness of nonverbal cues, potentially weakening data robustness. Fourth, social desirability bias may have led participants to offer socially acceptable rather than genuine responses. Furthermore, the pre-existing professional relationship between the research team and participants could have reduced the authenticity of disclosures, particularly regarding negative or sensitive views. We attempted to mitigate these biases by employing independent researchers, guaranteeing strict confidentiality and anonymity, and fostering a non-judgmental interview environment, yet their potential influence cannot be entirely eliminated.

## Conclusions

The experience of patients with TAVR and their caregivers in digital home-based cardiac rehabilitation is a dynamic process, with distinct barriers and facilitators across the pre-discharge, 1-month post-intervention, and 3-month post-intervention phases. For the pre-discharge stage, digital rehabilitation programs should integrate tailored hands-on device training and simplified visual educational materials to address patients’ confusion and caregivers’ insufficient capacity. In the 1-month phase, programs need to incorporate nurse-led remote support and community-based on-site follow-up to mitigate symptom management struggles and inaccessible medical services. For the 3-month phase, personalized rehabilitation adjustments and caregiver respite resources are critical to address adherence fluctuations. Policymakers and healthcare systems should prioritize standardizing TAVR-specific digital platforms with geriatric-friendly designs and social support features, while ensuring sufficient healthcare professionals and accessible community resources to sustain rehabilitation effectiveness.

## Supplementary Information

Below is the link to the electronic supplementary material.

Supplementary Material 1

Supplementary Material 2

## Data Availability

Regarding data availability, the corresponding author can provide the data that substantiates the results of this interview when requested reasonably.
