# Supplementary material for: Rehabilitation needs and adherence among TAVR patients and their caregivers during digital home-based cardiac rehabilitation: a qualitative longitudinal study
Source: BMC Nurs. 2026 Apr 6;25:456. doi: 10.1186/s12912-026-04631-x (PMC13188331; doi:10.1186/s12912-026-04631-x)
Supplement: Supplementary file 1 — Supplementary Material 1 [file 12912_2026_4631_MOESM1_ESM.docx]

**Appendix A Implementation version of home-based cardiac rehabilitation program for patients with TAVR**

| Step | Time | Participants | Content |
| --- | --- | --- | --- |
| Home-Based Cardiac Rehabilitation Assessment for TAVR Patients | 5 to 6 days before discharge | Multidisciplinary team, patients and their caregivers | 1. Establish a multidisciplinary cardiac rehabilitation team consisting of physicians, head nurses, staff nurses, nursing postgraduates, and rehabilitation therapists. 2. Inform patients and their caregivers about the objectives, benefits, and risks of the home-based rehabilitation program, and obtain their informed consent. 3. Invite patients and their caregivers to join the WeChat group for home-based rehabilitation, follow the official WeChat public account, and register on the WeChat mini-program for home-based cardiac rehabilitation. |
|  | 3 to 4 days before discharge |  | 1. For patients who voluntarily participate in the study, the team dynamically conducts home-based cardiac rehabilitation risk assessments on them. 2. The multidisciplinary team dynamically assesses the patient's general information, medical history, laboratory tests, valve function, complications and comorbidities, deep vein thrombosis, frailty, motor and cognitive functions, flexibility and balance, activities of daily living, digital literacy, and network environment. 3. The assessment levels are classified into three grades: high, medium, and low, and patients who do not meet the inclusion and exclusion criteria are excluded. 4. Collect patients' data in a one-on-one manner and group the patients according to random numbers. 5. Distribute printed and electronic versions of the home-based rehabilitation health management manual and home-based rehabilitation management log to patients in the experimental group. |
|  | 1 to 3 days before discharge | Nurses, patients and their caregivers | 1. Nurses explain knowledge related to home-based cardiac rehabilitation to patients in the experimental group; teach them how to use the home-based rehabilitation log and manual; instruct them on the identification of dangerous symptoms during home-based rehabilitation and first-aid knowledge; guide patients in the experimental group and their caregivers to use the home-based cardiac rehabilitation system; and elaborate on the methods of conducting aerobic exercise at home. 2. Nurses explain the contents of the manual and precautions to patients in the experimental group and their caregivers, covering aspects such as exercise, medication, nutrition, and psychology. |
| Home-Based Exercise Management for TAVR Patients | 1 to 2 days before discharge | Multidisciplinary team and patients | 1. Nurses push home exercise videos to patients in the experimental group and their caregivers via the cardiac rehabilitation system. The content includes the type, intensity, frequency and duration of exercise, as well as adverse reactions during exercise and corresponding countermeasures. |
|  | The 1st week after the intervention starts | Nurses and patients | 1. Nurses conduct a monthly telephone follow-up with patients in the experimental group to understand their rehabilitation status and adjust the rehabilitation plan in a timely manner based on the patients' conditions. 2. Based on the digital system, nurses send exercise reminders (twice a day, two days a week) and health management reminders to patients in the experimental group every week. |
|  | Weeks 1 to 12 after the intervention starts | Nurses and patients | 1. Patients in the experimental group shall fill in the exercise log in a timely manner after completing their exercise. The log shall include information such as blood pressure and heart rate before and after exercise. |
| Cultivation of Home-Based Rehabilitation Motivation for TAVR Patients | The 1st week after the intervention starts | Nurses and patients | 1. Combined with in-hospital health education, nurses push videos about the concept, benefits and health outcomes of home-based rehabilitation to patients via the cardiac rehabilitation system. 2. Combined with in-hospital health education, nurses push a video about the incidence rate, influencing factors and harms of valvular heart disease once via the cardiac rehabilitation system. 3. Develop a personalized rehabilitation plan based on the patient's preference (choosing one from brisk walking and housework), and the patient shall perform activities within the range of 12-14 points on the Borg Scale. Aerobic exercise shall be done twice a week, with each session lasting 30-40 minutes. |
| Creation of a Home-Based Rehabilitation Environment for TAVR Patients | The 2nd week after the intervention starts | Nurses and patient caregivers | 1. Encourage patients in the experimental group and their caregivers to actively engage in discussions in the home-based rehabilitation group and actively participate in online cardiac rehabilitation lectures. |
|  | Follow-up every Sunday | Nurses and patients | 1. The rehabilitation team helps patients in the experimental group identify and overcome barriers affecting health-related behaviors. For patients who live alone, have low educational levels, or face other similar circumstances, the team provides them with health education and emotional support. |
| Home-Based Risk Factor Management for TAVR Patients | The 3rd week after the intervention starts | Nurses and patients | 1. Based on the cardiac rehabilitation system, nurses push videos and articles for health education on post-operative risks to patients in the experimental group and their caregivers, covering knowledge related to valvular heart disease, mental health, and sleep management. |
|  | Weeks 1 to 4 after the intervention starts | Nurses, patients and their caregivers | 1. Nurses educate the caregivers of patients in the experimental group on the importance of family support, and encourage the caregivers to provide support to the patients through forms such as emotional support, rehabilitation assistance, dietary management, companionship and communication, rehabilitation supervision, and family environment improvement. 2. Nurses send a set of home-based rehabilitation and health management information to patients in the experimental group and their caregivers at a fixed time every week. |
| Home-Based Symptom and Follow-Up Management for TAVR Patients | The 4th week after the intervention starts | Nurses, patients and their caregivers | 1. Based on the cardiac rehabilitation system, nurses push videos on the prevention, symptoms, and management measures of long-term complications after TAVR. 2. Based on the cardiac rehabilitation system, nurses push videos about home-based follow-ups, as well as videos and articles covering the purpose, timing, importance, examination content and precautions of follow-ups. 3. Nurses regularly remind patients of their follow-up appointments via the cardiac rehabilitation system or phone calls. |
