# Supplementary material for: Rehabilitation needs and adherence among TAVR patients and their caregivers during digital home-based cardiac rehabilitation: a qualitative longitudinal study
Source: BMC Nurs. 2026 Apr 6;25:456. doi: 10.1186/s12912-026-04631-x (PMC13188331; doi:10.1186/s12912-026-04631-x)
Supplement: Supplementary file 2 — Supplementary Material 2 [file 12912_2026_4631_MOESM2_ESM.docx]

**Appendix B**

**Interview outline**

Note: The interviews began with the facilitator asking general health-related questions, followed by specific inquiries about the needs, facilitators, and barriers of digital home-based cardiac rehabilitation. questions may be asked in a different order, or some omitted or added, depending on the participants’ responses. Follow-up questions were included to encourage participants to describe their perceptions as much as possible.

**Appendix B1** Semi-structured interview questions for patients approached discharge.

| Category | Interview questions |
| --- | --- |
| Caregivers of patients undergoing TAVR | What do you think about the digital home-based cardiac rehabilitation? (Advantages and disadvantages)  What are your main concerns or needs regarding your loved one’s discharge from the hospital?  How do you plan to manage their care at home? Are there any areas where you feel you need more assistance? |
| Patients undergoing TAVR | How did you feel during your hospital stay post-TAVR?  Do you feel that you received enough information about the TAVR procedure and recovery process?  What are your main concerns or needs as you prepare to leave the hospital?  Do you know how to carry out digital home-based cardiac rehabilitation after discharge? what additional support would help?  What kind of help do you hope the medical staff can provide for you?  What are the advantages of digital home-based cardiac rehabilitation in your opinion?  Would you like to participate in the home-based cardiac rehabilitation? Do you plan to consistently engage in Cardiac rehabilitation? |

**Appendix B2** Semi-structured interview questions for one-month after the intervention.

| Category | Interview questions |
| --- | --- |
| Caregivers of patients undergoing TAVR | How have your caregiving duties evolved since the patient was discharged?  Have there been any challenges or changes in your caregiving role that you didn’t anticipate?  What kind of support or resources have you found most useful in managing your caregiving role?  Are there specific areas where you feel you need additional support or resources?  Have you encountered any specific challenges or obstacles in managing your heart recovery at home? How have you addressed or overcome these challenges?  Is there anything else you would like to share about your recovery experience or caregiving role?  How do you plan to manage ongoing caregiving responsibilities in the coming months? |
| Patients undergoing TAVR | How have you been feeling since your discharge from the hospital one month ago?  Can you describe your experience with the heart recovery process over the past month?  - How have you been monitoring and managing your symptoms and overall health?  What barriers or difficulties have you faced in terms of accessing care or following medical recommendations? How have these barriers impacted your recovery or caregiving?  Is participating in the digital home-based cardiac rehabilitation challenging for you? |

**Appendix B3** Semi-structured interview questions for three months after the intervention.

| Category | Interview questions |
| --- | --- |
| Caregivers of patients undergoing TAVR | What obstacles and factors do you think exist for patients to participate in home-based cardiac rehabilitation?  What factors have influenced your ability to support the patient effectively?  Are there any specific challenges you have encountered in your caregiving role?  Can you describe your current role in supporting the TAVR patient with their recovery?  How has your caregiving role evolved over the past three months?  Is there anything else you would like to share about your experience with heart recovery or caregiving?  Do you have any suggestions for improving support and resources for TAVR patients and their caregivers? |
| Patients undergoing TAVR | What specific steps have you taken to adhere to your heart recovery plan since discharge?  How consistently have you been able to follow your prescribed medication, exercise, and dietary recommendations?  How are you feeling now, three months after your discharge?  Can you describe your overall experience with heart recovery over the past three months?  What rehabilitation needs are not being met during your home-based cardiac rehabilitation?  How have these barriers affected your or the patient’s ability to adhere to the recovery plan? |
